# The After-Effects of Cerebro-Spinal Meningitis

**Published:** 1909-09-25

**Authors:** 


### THE AFTER-EFFECTS OF CEREBRO-SPINAL MENINGITIS.

Of the four chief forms of acute meningitis—suppurative, basal, posterior basal, and epidemic cerebro-spinal—the last two are probably identical in kind, though they may differ from one another somewhat in type. They have an altogether different prognosis from the other two, for whereas all cases of suppurative, and almost all of basal or tuberculous meningitis prove fatal, about one-third or more of the cases due to the meningo-coccus recover. Out of eighty-two cases recently collected by Cohn, fifty died in hospital, two died after leaving hospital, three were lost sight of, and twenty-seven recovered and were traced for some while afterwards. The latter could be subdivided, according to their severity, into eight mild, fifteen medium, and four severe cases.

Recovery in the mild cases was complete, nothing whatever remaining to distinguish these children from others who had not been ill at all.

Some of the cases of medium severity recovered completely, either without or with a period of delay. In a fair proportion, however, there were permanent after-effects of one sort or another. Out of the fifteen cases in this group many complained of headache afterwards, either on exertion or upon trying to read or write, or on stooping; in three patients there was obvious loss of memory, and in two much difficulty in concentrating attention; in three others there was sufficient weakness of the arms or legs or both to make any serious exercises impossible. It was difficult to say whether this paresis would be quite permanent, however, for in at least two other cases there had been marked paralysis of the extremities and of ocular muscles on discharge from hospital, and yet these had recovered entirely later on.

Of the severe cases that recovered, two did so completely—a very encouraging fact. The remaining two remained deaf and had facial palsy, one also suffering from paresis of one arm for a long time, though the limb ultimately got well. The wonderful way in which all the exuded serum and lymph in the meninges may be reabsorbed was shown by the post-mortem examination in one of the severe cases that died of diphtheria and heart failure a year after recovery from cerebro-spinal meningitis. The only changes to be found within the cranium were some adhesions between the pia-arachnoid and the bone at the level of the anterior fontanelle.

